# Impact of Hypertension on the Association of BMI with Risk and Age at Onset of Type 2 Diabetes Mellitus: Age- and Gender-Mediated Modifications

**DOI:** 10.1371/journal.pone.0095308

**Published:** 2014-04-17

**Authors:** Arshad Mohamed Channanath, Bassam Farran, Kazem Behbehani, Thangavel Alphonse Thanaraj

**Affiliations:** Dasman Diabetes Institute, Dasman, Kuwait; Daping Hospital, Third Military Medical University, China

## Abstract

**Aims:**

Given that BMI correlates with risk of Type 2 diabetes mellitus (T2DM), and that hypertension is a common comorbid condition, we hypothesize that hypertension augments significantly the impact of obesity on T2DM onset.

**Methods:**

We obtained data on T2DM in Kuwaiti natives from Kuwait Health Network Registry. We considered 1339 comorbid individuals with onset of hypertension preceding that of T2DM, and 3496 non-hypertensive individuals but with T2DM. Multiple linear regressions, ANOVA tests, and Cox proportional hazards models were used to quantify the impact of hypertension on correlation of BMI with age at onset and risk of T2DM.

**Results:**

Impact of increasing levels of BMI on age at onset ot T2DM is seen augmented in patients diagnosed with hypertension. We find that the slope of the inverse linear relationship between BMI and onset age of T2DM is much steep in hypertensive patients (−0.69, males and −0.39, females) than in non-hypertensive patients (−0.36, males and −0.17, females). The decline in onset age for an unit increase of BMI is two-fold in males than in females. Upon considering BMI as a categorical variable, we find that while the mean onset age of T2DM in hypertensive patients decreases by as much as 5–12 years in every higher BMI categories, significant decrease in non-hypertensive patients exists only when severely obese. Hazard due to hypertension (against the baseline of non-hypertension and normal weight) increases at least two-fold in every obese category. While males have higher hazard due to hypertension in early adulthood, females have higher hazard in late adulthood.

**Conclusion:**

Pre-existing condition of hypertension augments the association of BMI with Type 2 diabetes onset in both males and females. The presented results provide health professionals directives on the extent of weight-loss required to delay onset of Type 2 diabetes in hypertensive versus non-hypertensive patients.

## Introduction

Associations between obesity and incident Type 2 diabetes mellitus (T2DM) have been extensively studied [1], [2], [3], [4], [5], [6], [7], [8], [9]. Inverse linear relationships have been observed between body mass index (BMI) and age at onset of T2DM [10], [11], [12]. It has been shown with European population that T2DM can be diagnosed at lower BMI in men than in women [13]. Furthermore, the magnitude of association between weight gain and risk of T2DM can be different in different age groups; for example, weight gain in early adulthood is linked to a higher risk and early onset of T2DM than weight gain in middle adulthood (40 and 55 years of age) [14]. The above-cited studies have not considered hypertension as a confounder, despite the observations that hypertension is a common comorbid condition with T2DM [11], that presence of hypertension acts as a significant predictor in prognostic and diagnostic models for onset of T2DM [15], [16], [17], [18], and that people with even pre-hypertension show a higher risk of developing T2DM than those with normal blood pressure [19]. Blood pressure levels are themselves correlated with BMI [20], suggesting the possibility that significant interactions may exist between BMI and hypertension in determining the risk of incident T2DM. However, to the knowledge of the authors, the interaction between hypertension and BMI has not been considered in studies on risk of T2DM due to obesity. We aim to quantify the interactions between hypertension and BMI on T2DM onset and attempt to quantify those interactions so as to appropriately tailor diabetes screening and prevention measures.

Clinical practice guidelines that help in prevention of T2DM present recommendations on dealing with risk factors. The risk factors are of at least three types: Modifiable risk factors that include obesity (BMI), hypertension, lipid disorders, depression, hyperglycemia, unhealthy diet, and low physical activity; non-modifiable risk factors that include age, family history of diabetes, ethnicity, and low-birth weight; and environmental risk factors that include low socioeconomic status, cultural constraints, religious practices, and distress. Impact due to risk factors on onset of T2DM is exerted through a combinatorial interplay of risk factors from all of the above-mentioned three types. Delineation of this interplay will help in developing further the current guidelines on diabetes screening and prevention. Using a data set of nationwide trends, from the Kuwait Health Network Registry, on age at onset of T2DM, BMI, gender, and hypertension status, we seek to explore how the pre-existence of hypertension modifies the impact of BMI on risk & onset of T2DM. More specifically, we quantify the collective associations among age, gender, hypertension status and BMI that define the onset age and risk for T2DM.

## Materials and Methods

The study was approved by the Ethics Committee at Dasman Diabetes Institute, Kuwait. The patient records/information was anonymized and de-identified prior to analysis.

The study uses nation-wide data extracted from Kuwait Health Network (KHN) Resource [11], [16] on native patients visiting primary health care centers and hospitals that the Ministry of Health operate in five health regions (namely Hawally, Farwaniya, Jahara, Ahmadi, and Capital) of Kuwait. The derivation of the data set is as described in [16]. The data set contains information on a total of 270172 participants that visited the hospitals. Each of these 270172 unique participants is assigned a unique file containing multiple records (a record corresponds to a single visit to the hospital). A participant can have multiple visits to the hospital and hence a participant corresponds to one or more hospital records.

The participants include both natives and expatriates - Kuwaiti natives form 55%; Asian expatriates (largely from the Indian subcontinent) form 24%; Arab expatriates (largely from Egypt) form 16%; and expatriates from other countries form 5%. 74134 of the 270172 participants are diagnosed with T2DM – ethnicity composition of the T2DM population is: Kuwaiti natives: 51%; Asian expatriates: 29%; Arab expatriates: 15%; and expatriates from other continents: 5%.

From this nation-wide data, we carve out the following two data sets, to study the impact of hypertension on the association of BMI with risk and age at onset of T2DM, considering only the Kuwaiti natives: (i) Comorbid patients with diagnosis for hypertension preceding T2DM and with complete record of gender, BMI, and age at onset of diabetes/hypertension (n = 1339); and (ii) T2DM patients with no incidence of hypertension and with complete record of gender, BMI and age at onset of T2DM (n = 3496).

There are many reasons for the drop in the number of participants in the final list (from 40774 Kuwaiti-native T2DM patients to 4835). The primary source of data is national registry of health records. The Kuwait National Health Network integrates data from five health regions (Hawally, Farwaniya, Jahara, Ahmadi, and Capital) of Kuwait; the data resources that are integrated are extracted from the information systems at primary health centers, and at the Ministry of Health hospitals (as stored in their Laboratory Information systems and Health Information systems), and at administrative offices of Public Administration of Civil Information; integration of all relevant information from these multiple sources is still an ongoing process and hence certain participants in the registry have incomplete integration. Secondly and most importantly, we make sure that the considered participants in the final data set are not one-time or rare visitors to the clinics/hospitals; they need to be regular visitors before and after the diagnosis for diabetes and for hypertension; we require there exists at least 3 visits in the year prior to the diagnosis date. Of the diabetic patients, only those that developed diabetes after the onset of hypertension are considered. Further, we carry out sanity checks to make sure that the recorded values for the different data items are consistent with one another.

Age-wise distributions are studied by considering the following three age groups: 20–40 years (young adulthood), 40–60 years (middle adulthood), and ≥60 years (late adulthood). For categorizing patients according to BMI, the classification system approved by the WHO is used: normal weight (BMI  = 18.5–24.9 kg/m^2^), overweight (25.0–29.9 kg/m^2^), mildly obese (30.0–34.9 kg/m^2^), moderately obese (35.0–39.9 kg/m^2^), and severely obese (≥40 kg/m^2^). The ‘normal weight’ category is considered as the reference BMI category. In those instances where BMI measurements at the time of diagnosis for diabetes are unavailable, the nearest measurements (made within a year of diagnosis) are used.

Multiple Linear Regression models are used to examine the association between BMI and age at onset of T2DM. ANOVA (ANalysis Of VAriance), with Tukey's post hoc Honestly Significant Difference test, is performed to evaluate gender-specific differences in mean onset ages in various BMI categories. Hazard ratios (HR) that evaluate the risk of T2DM in hypertensive patients of various BMI categories (against the baseline characteristics of non-hypertensive patients of normal weight category) are calculated using Cox proportional hazards regression models. The Hazard ratios are calculated for each of the three age groups and are corrected for gender.

Data analyses are performed using the R Project for Statistical Computing (http://www.r-project.org/). Results are considered statistically significant at p-value <0.05. Continuous data are expressed as mean and standard deviation, while categorical data are expressed as absolute subject numbers and percentages.

## Results

### Prevalence of T2DM, hypertension and comorbidity – natives versus expatriates

#### Prevalence of Type 2 diabetes

27% of the 270172 participants are seen diagnosed with Type 2 diabetes. Crude prevalence of Type 2 diabetes among Asian expatriates (at 33.25%) is seen higher than among Kuwaiti natives (at 25.4%) and Arab expatriates (at 26.6%). Considering the age range of 30–60 years, the age-specific prevalence of Type 2 diabetes among all hospital visitors is 31.5%, and ethnic-wise prevalence is 30.3% (Kuwaiti natives), 34.6% (Asian expatriates), and 28.7% (Arab expatriates).

#### Prevalence of hypertension

Around 31% of the participants in Kuwait are seen clinically diagnosed for hypertension. Ethnic differences are seen in the crude prevalence (Kuwaiti natives at 28%; Asian expatriates at 37%; Arab expatriates at 34%). However, considering only the 30–60 age group, the age-specific prevalence of hypertension is 36% (Kuwaiti natives), 39% (Asian expatriates), and 38% (Arab expatriates).

#### Prevalence of comorbidity

We see that 41% of Type 2 diabetic patients are also diagnosed with hypertension (and that 36% of the hypertensive population is also diagnosed with diabetes). Age-specific prevalence of affliction by both hypertension and diabetes (age range: 30–60 years) is seen at 11% irrespective of ethnicity. In 70% of comorbid patients, Type 2 diabetes precedes hypertension.

### Descriptive statistics on data sets

The descriptive statistics for the two data sets of T2DM onset in hypertensive patients, and T2DM onset in non-hypertensive patients (see Materials and Methods) are summarized in Table 1 and detailed in Table S1. The mean duration of hypertension before the onset of diabetes is seen at 3.96±2.8 years. Prior to the onset of diabetes, the mean duration of visiting the hospital system is seen at 2.82±2.6 years (in the case of data set on diabetes in non-hypertensive) and 5.30±3.25 years (in the case of data set on diabetes in hypertensive). The mean BMI of the participants in each of the case and control data sets is in the range of 33–35 kg/m^2^; the mean BMI of the patients with diabetes and hypertension (at 34.9±6.9) is higher than that of the patients with only diabetes but no hypertension (at 33.0±6.5) (p<0.01). The mean blood pressure readings of the patients with diabetes and hypertension (at SP  = 148.45±20.39; DP  = 91.49±11.82) are higher than those of the patients with only diabetes but no hypertension (at SP  = 127.25±15.35; DP  = 80.56±8.71) (p<0.01 in both the cases of SP and DP).The mean HbA1C of the participants in each of the case and control data sets is in the range of 8.3–9.4. 44% of the patients with diabetes and hypertension as opposed to 33% of the patients with diabetes but no hypertension are ‘moderately’ or ‘severely’ obese. The mean BMI of females is higher than that of males (males = 31.8±6.3, females = 35.1±6.8, p<0.01). The mean age at onset of hypertension or T2DM is lower in males than in females (hypertension: males = 51.9±12.3, females = 53.7±11.3 years, p<0.01) and T2DM (males = 48.3±11.4, females = 50.2±11.0 years, p<0.01). The mean onset age of T2DM (at 51.7±11.9, males; 54.5
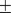
10.50, females) in the considered comorbid (i.e. T2DM in hypertensive) patients is higher than that in non-hypertensive patients (at 46.7
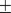
11.3, males; 47.9
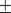
10.8, females).

**Table 1:** Summary of descriptive statistics of the data sets used in the study.

	T2DM in hypertensive patients (n = 1339)	T2DM in non-hypertensive patients (n = 3496)	p-value@
**Mean BMI in kg/m^2^**	34.9±6.94	33.0±6.55	p<0.001
**Mean Blood pressure values in mmHg**	SP = 148.45±20.39	SP = 127.25±15.35	p<0.001
	DP = 91.49±11.82	DP = 80.56±8.71	p<0.001
**Mean HbA1C values in %**	8.3±2.3	9.4±2.4	P<0.001
**Mean duration of hypertension before onset of T2DM in years**	3.96±2.8	NA	NA
**Mean duration of registration before the onset of hypertension in years**	1.20±1.82	NA	NA
**Mean duration of registration before the onset of T2DM in years**	5.30±3.25	2.82±2.6	P<0.001
**Mean onset age of T2DM in years**	53.45±11.00	47.34±11.04	P<0.001
**Mean onset age of hypertension in years**	49.29±11.00	NA	NA

@, The mean values presented in the previous two columns are compared using t-test.

### Association of BMI (as continuous risk) with age at onset of T2DM in hypertensive and non-hypertensive patients

Multiple linear regression models with BMI & gender as predictor variables and age at onset of T2DM as outcome variable lead to the following approximations:

**Onset age  = 73.03− [0.52× (BMI)] − [4.30× (Gender being male)]**. (T2DM in hypertensive patients; n = 1339).

**Onset age  = 61.80− [0.33× (BMI)] −2.90× [(Gender being male)]**. (T2DM in non-hypertensive patients; n = 3496).

The above models indicate that age at onset of T2DM is inversely correlated with BMI in both hypertensive and non-hypertensive patients; the slope of the association is more steep in hypertensive patients at m = −0.52 (95% CI [−0.59, −0.43]) than in non-hypertensive patients at m = −0.33 (95% CI [−0.37, −0.29]). Gender being male reduces the onset age for T2DM in hypertensive patients by 4.30 years compared to 2.90 years in non-hypertensive patients.

The higher intercept value of 73.03 seen in the case of T2DM in hypertensive patients against 61.80 seen with T2DM in non-hypertensive patients is due to the study design and the nature of the constructed data sets; as per the descriptive statistics of the data sets (see Table 1), the mean age at onset of T2DM is higher in hypertensive patients (by 5 years in the case of males and by 7 years in the case of females) than in non-hypertensive ones.

In order to quantify the effect of gender on the association of BMI with onset age of T2DM, we rebuild the above models by introducing an interaction term between gender and BMI as an additional variable. The models lead to the following approximations:

**Onset age  = 68.65− [0.39× (BMI)] − [(0.30× Gender being male) × (BMI)] + [5.89× (Gender being male)]** (T2DM in hypertensive patients; n = 1339).

**Onset age  = 54.09− [0.17× (BMI)]− [(0.19× Gender being male) × (BMI)] + [4.43× (Gender being male)]** (T2DM in non-hypertensive patients; n = 3496).

These translate into the following gender-wide approximations:

**(T2DM in hypertensive patients)**:

Onset age  = 74.54–0.69 (BMI): Males

Onset age  = 68.65–0.39 (BMI): Females

**(T2DM in non-hypertensive patients)**:

Onset age  = 58.52–0.36 (BMI): Males

Onset age  = 54.09–0.17 (BMI): Females

**Figure 1:**
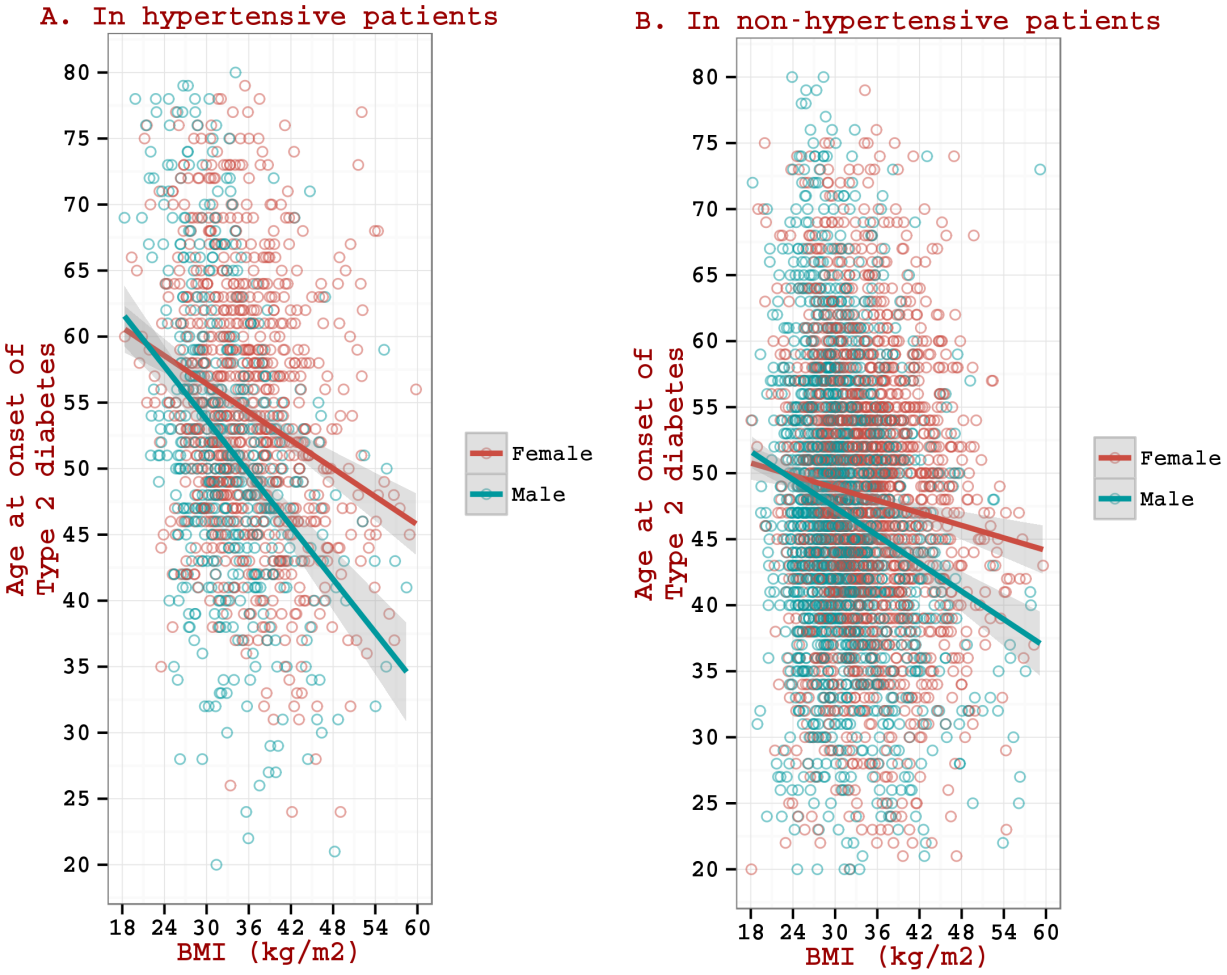
Multiple linear regression models (with introducing an interaction term between gender and BMI as an additional variable) for association between BMI and age at onset of Type 2 diabetes in hypertensive and non-hypertensive patients.

With the revised models (Figure 1), the slopes are seen even more significantly different (p<0.001) between males and females in both the hypertensive patients (at m = −0.69, 95% CI [−0.85, −0.53], for males and m = −0.39, 95% CI [−0.49, −0.29], for females) and the non-hypertensive patients (at m = −0.36, 95% CI [−0.47, −0.25], for males and m = −0.17, 95% CI [−0.25, −0.10], for females). The difference in age at onset of T2DM in males versus females becomes increasingly prominent with increasing levels of BMI (see Figure 1).

### Pre-existence of hypertension is not a bystander but significantly modifies the association between BMI and onset age of T2DM

In order to test whether association between BMI and age at onset of T2DM is indeed influenced by pre-existing condition of hypertension, we perform a test of interaction between hypertension status and BMI in a regression analysis by pooling together the two data sets: (with gender and hypertension status as covariates, and age at onset of T2DM as dependent parameter):

Ŷ = 57.45−0.27 (x BMI) −2.72 (x Gender being male) −0.23 (BMI * Hypertension-status) +14.24 (x Hypertension-status) (n = 4835).

The regression coefficient associated with the interaction term between BMI and hypertension status in the above model is seen statistically significant (slope  = −0.226; *P<0.001*). The other coefficients are also seen statistically significant (*P<0.001*).

### Reduction in onset age of T2DM in increasingly obese BMI categories (BMI as threshold risk) of hypertensive patients

We further examine, using ANOVA with Tukey's post hoc Honestly Significant Difference tests, the changes in the onset age of T2DM across different BMI categories (Table 2). As BMI increases from normal weight to increasingly obese categories (namely overweight, moderately obese, and severely obese), the mean onset age decreases considerably in hypertensive patients by 5 (overweight category) to 12 years (severely obese category). In the case of non-hypertensive patients, only severely obese category shows significant differences.

**Table 2:** Decrease in the mean age at onset of T2DM across different BMI categories in hypertensive and non-hypertensive patients.

BMI categories	Overweight		Mildly Obese		Moderately Obese		Severely Obese	
	Hypertensive	Non-hypertensive	Hypertensive	Non-hypertensive	Hypertensive	Non-hypertensive	Hypertensive	Non-hypertensive
**Normal**	5.4 *(1.6,9.3) p<0.005*	0.6* *(−1.2, 2.6) p = 0.9*	6.7 *(3.0–10.5) p<0.001*	0.6* *(−1.3,2.4) p = 0.9*	9.1 *(5.3,12.9) p = 0*	1.2* *(−0.8,3.2) p = 0.4*	12.1 *(8.2, 15.9) p = 0*	3.3 *(1.2, 5.4) p<0.001*
**Overweight**	-	-	1.3* *(0.8, 3.4) p = 0.4*	1.2* *(0.0,2.5) p = 0.05*	3.7 *(1.5–5.9) p<0.001*	1.9 *(0.5,3.3) p<0.05*	6.6 *(4.3, 8.9) p = 0*	4.0 *(2.5,5.6) p = 0*
**Mild Obese**	-	-	-	-	2.4 *(0.3–4.4) p<0.05*	0.7* *(−0.7,2.1) p = 0.7*	5.2 *(3.1, 7.4) p = 0*	2.8 *(1.3,4.3) p<0.001*
**Moderate Obese**	-	-	-	-	-	-	2.9 *(0.7–5.1) p<0.005*	2.1 *(0.43,3.8) p<0.05*

*, these values are not significant. P-value ≥0.05

**Figure 2:**
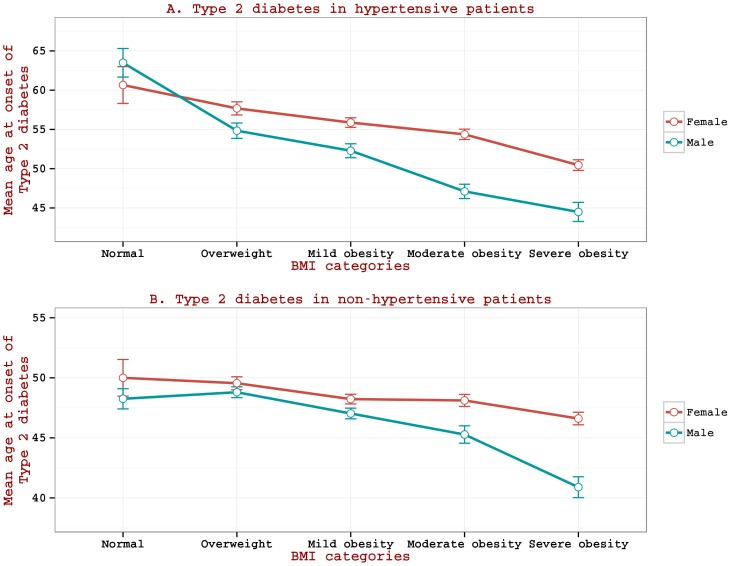
Mean age at onset of Type 2 diabetes in different BMI categories. (A) in hypertensive patients; (B) in non-hypertensive patient.

Examination of the effect of gender on the onset age (as increasingly obese categories are considered) illustrates that the onset age is consistently lower in males than in females for every BMI category (Figure 2). The gender specificity is significantly evident in hypertensive patients; the difference in onset ages between females and males in the moderately obese and severely obese categories (as compared to normal weight categories) are 7.3 years (p<0.001) and 6.0 years (p<0.001) in hypertensive patients (Figure 2.A.) as opposed to 2.8 years (p<0.001) and 5.7 years (p<0.001) in non-hypertensive patients (Figure 2.B.).

### Differences between age at the onset of T2DM in hypertensive group and non-hypertensive group

**Figure 3:**
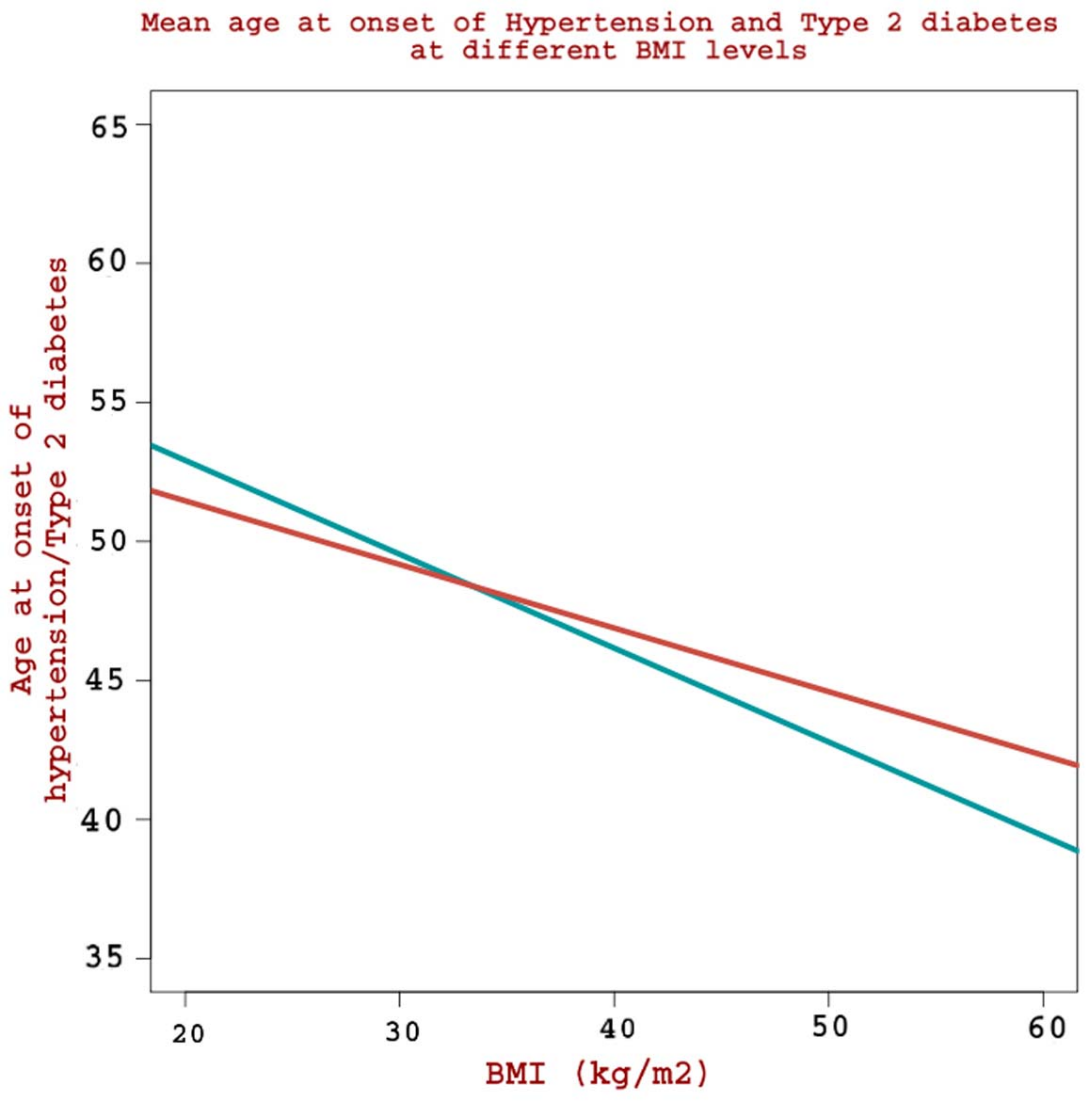
Mean age at onset of Hypertension and T2DM at different BMI levels.

Examination of Figures 1 and 2 indicates that age at onset of T2DM in hypertensive group (particularly in patients with lower BMI values) is higher than that in non-hypertensive group. Age at onset of T2DM in the cohort of hypertensive patients has a baseline reference to age at onset of hypertension, as this data set requires onset of hypertension prior to T2DM (while such a situation does not exist in the cohort of non-hypertensive patients). To investigate as to why a higher onset age of T2DM is seen in the hypertensive cohort, we examined the mean age at onset of hypertension at various BMI levels (as measured at the time of hypertension onset) in a cohort of hypertensive patients (n = 1516) with no co-occurrence of T2DM (Figure 3). Age at onset of hypertension is seen higher at lower BMI values (e.g. onset age at BMI = 30 is 51 years) and the drop in onset age per unit increase in BMI is very sharp (onset age at BMI = 50 is 44 years) with the rate of drop at −0.41. Figure 3 also presents mean age at onset of T2DM at various BMI levels (as measured at the time of T2DM onset) in a cohort of T2DM patients (n = 3496) with no co-occurrence of hypertension; we observe that while age at onset of hypertension is generally higher than that of T2DM at lower BMI values, it is generally lower than that of T2DM at higher BMI values. At around a BMI of 30 Kg/m^2^, the mean age at onset of hypertension becomes equal to that of T2DM. The rate of drop in onset age of hypertension per unit increase in BMI is very sharp (at slope  = −0.41) as compared to that of T2DM at −0.22.

### Hazard ratios for developing T2DM in hypertensive patients of increasingly obese categories

We evaluate Cox proportional Hazard ratios (HR) for the risk of T2DM in hypertensive patients of different BMI categories against the baseline characteristics non-hypertensive patients of normal weight categories; the models are corrected for gender. The differences in such hazard ratios in different categories of BMI are given in Table 3 for the three age groups of early adulthood, middle adulthood, and late adulthood. Considering the age group of middle adulthood (40–59 years), the data points out that in the case of hypertensive patients, the hazard increases (at least two-fold over the hazard in non-hypertensive normal weight patients) as increasingly obese categories are considered. Evaluation of differences in hazard ratio (HR) due to hypertension between males and females (Table 3, last column) indicates that males have higher risk in the age group of early adulthood while females are more at risk in the age group of late adulthood - in the age group of early adulthood, the such hazard ratios for males is 1.8 (95% CI [1.2, 2.61] p<0.01) times than that of females and in late adulthood it is 0.75 (95% CI [0.59, 0.95] p = 0.02).

**Table 3:** BMI-wide and Gender-wide differences in Hazard ratios for risk of developing T2DM in hypertensive patients (against baseline diagnosis in non-hypertensive patients from normal weight categories).

Age group (in years)	BMI-wide differences in hazard ratios, corrected for gender				Gender-wide differences in hazard ratios (Males versus Females)
	Overweight category	Mildly obese category	Moderately obese category	Severely obese category	
**20–39**	2.1* *(0.27, 16.04) p = 0.48; n = 192*	3.3* *(0.47, 26.07) p = 0.22; n = 210*	7.3* *(1.01, 54.27) p = 0.05; n = 181*	9.2 *(1.27, 67.33) p = 0.03; n = 196*	1.8 *(1.21, 2.61) p<0.01; n = 830*
**40–59**	1.6 *(1.05, 2.37) p = 0.03; n = 817*	2.3 *(1.53, 3.39) p<0.001; n = 1010*	2.6 *(1.74, 3.89) p<0.001; n = 642*	3.5 *(2.37, 5.36) p<0.001; n = 496*	1.1* *(0.94,1.25) p = 0.24*; *n = 3170*
**60 and above**	1.2* *(0.84, 1.84) p = 0.27; n = 249*	2.1 *(1.39, 3.02) p<0.001; n = 239*	2.1*(1.34, 3.17) p<0.001; n = 133*	2.6 *(1.61, 4.12) p<0.001; n = 81*	0.75 *(0.59, 0.95) p = 0.02*; *n = 786*

*, these values are not significant. P-value ≥0.05.

The sample sizes relating to observations on hazard ratios in the age groups of early (age group of 20–39 years) and late adulthood (age group of ≥60 years) are small. However, we can infer the following as indications of trends in risk modification due to age: Higher BMI levels attribute more risk of developing T2DM in lower age groups than in higher age groups - for example, being severely obese increases the hazard 9.20 fold (95% CI [1.27, 67.33] p = 0.03;) in hypertensive patients from the age group of 20–39 years (considering normal weight category of non-hypertensive patients from the same age group as baseline), as opposed to an increase of only 3.5 (95% CI [2.37, 5.36] p<0.001) fold in the age group of 40–59 and as opposed to an increase of only 2.6 (95% CI [1.61, 4.12] p<0.001) fold in the age group of ≥60 years.

## Discussion

Diabetes is fast becoming a global epidemic. According to the World Health Statistics 2012 from the WHO, one in 10 adults has diabetes, and one in three adults has hypertension. The International Diabetes Federation's 5th edition of the Diabetes Atlas (http://www.idf.org/diabetesatlas/5e/) projects that the number of people living with diabetes is expected to rise from 366 million in 2011 to 552 million by 2030, if no appropriate prevention measures are taken. The prevalence of T2DM and of the accompanying hypertension is an increasing problem in the states of the Co-operation Council for the Arab States of the Gulf (GCC) [11], [25], and these trends are set to continue. We find the age-specific prevalence of Type 2 diabetes among Kuwaiti natives as 30.3%, of hypertension as 28%, and of comorbidity as 11% (the corresponding values in the case of Asian expatriates living in Kuwait are: 31.5%, 36%, and 11% respectively). The chronic nature and long-term economic burden make diabetes one of the prototypical public health problems [21], [22]. Appropriate guidelines for risk factor stratifications that help in preventive interventions need to be prepared to contain the epidemic of diabetes, hypertension and the associated disorders.

Defining the relationship between body mass and risk of T2DM in the context of age, gender, and presence of other disorders such as hypertension is critical towards getting better understanding of underlying pathophysiological processes and in developing guidelines for risk factor stratifications. In the presented study, we examine the impact of hypertension on risk and age at onset of T2DM in obese patients and illustrate how this correlation is differentially modified by age and gender. The study points out the following observations:

- The slope of the negative correlation that age at onset of T2DM has on BMI is steeper in hypertensive patients than in non-hypertensive patients. The slopes are seen significantly different between males and females.
- In hypertensive patients, every categorical change in obesity levels leads to significant differences in the mean onset age for T2DM. However, in non-hypertensive patients, no significant change is seen in the onset age for T2DM until the severely obese category is reached. The onset age in every BMI category is consistently lower in males than in females; the difference is predominant in hypertensive patients than in non-hypertensive patients.
- Two-fold increase in hazard could be seen due to hypertension for every categorical change in BMI. Furthermore, it is seen that male hypertensive patients are at higher risk in the younger age group of 20–39 years while female hypertensive patients have a higher risk in the higher age group of ≥60 years.

Addressing diabetes is a major priority for health providers worldwide given the vast global prevalence and its severe complications including amputations and heart disease. Positive impact of weight reduction programmes or bariatric surgeries on prevention and treatment of T2DM has been recognized [23], [24]. The effect of BMI on the risk of and onset age for T2DM is confounded by gender, age, and presence of other disorders such as hypertension. Hypertension, which by itself is a risk factor for T2DM [16], shares many predisposing factors with T2DM. In this article, we report the collective associations among hypertension, age, gender, and BMI in defining the onset of incident T2DM. It is expected that the presented results provide important risk stratifications that are crucial in drawing guidelines for prevention and treatment of T2DM.

### 

#### Limitations of the study

Absence of assessment due to residual confounding by dietary and lifestyle factors, absence of information on antihypertensive medication, and absence of assessment of other measures of adiposity should be considered as limits in interpreting the results.

#### Impact of the observed findings on clinical practice

Various organizations, such as American Diabetes Association, Canadian Diabetes Association, Diabetes UK, and International Diabetes Federation have made available practice guidelines for prevention, intervention, and treatment of diabetes. All these guidelines invariably advise on maintaining healthy BMI. However, quantification of reductions in risk or delay in onset age by weight loss in patients stratified by presence or absence of hypertension in combination with other risk factors of age and gender is missing in these guidelines. The observed results in this study help fill this gap: an increase in BMI by one unit (1 Kg/m2) leads to early onset of T2DM by 0.69 years (in hypertensive males) and 0.39 years (in hypertensive females) as opposed to 0.36 years (in non-hypertensive males) and 0.17 years (in non-hypertensive females); and motivating severely and mildly obese hypertensive patients to lose weight until they reach normal healthy levels can delay the onset of T2DM by as much as 12 and 6.7 years, respectively.

## Conclusion

The results presented here help stratify people at the highest risk of acquiring Type 2 diabetes. In particular, individuals with hypertension appear to be more impacted by weight gain than those without hypertension. The presented results provide health professionals that monitor, support and care for obese people, directives on the extent of required weight-loss (for preventing or delaying onset of Type 2 diabetes) in accordance with hypertension diagnosis, gender and age.

## Supporting Information

Table S1 **Descriptive statistics of the data sets used in the study.**
(DOCX)
