# Supplementary material for: Impact of Hypertension on the Association of BMI with Risk and Age at Onset of Type 2 Diabetes Mellitus: Age- and Gender-Mediated Modifications
Source: PLoS One. 2014 Apr 17;9(4):e95308. doi: 10.1371/journal.pone.0095308 (PMC3990699; doi:10.1371/journal.pone.0095308)
Supplement: Table S1 — Descriptive statistics of the data sets used in the study. (DOCX) [file pone.0095308.s001.docx]

**Supporting Information**

**Table S1: Descriptive statistics of the data sets used in the study.**

|  | **T2DM in hypertensive patients (n=1339)** | **T2DM in non-hypertensive patients (n=3496)** | **p-value**^@^ |
| --- | --- | --- | --- |
| **Gender distribution** | | | |
| Male | 37.6% (n=503) | 46.9% (n=1639) | p < 0.001 |
| Female | 62.4% (n=836) | 53.1% (n=1857) | p < 0.01 |
| **Age-wide distribution (in years)** | | | |
| 0-19 | 0.3% (n=4) | 1.3% (n=45) | p<0.001 |
| 20-39 | 8.1% (n=108) | 20.7% (n=722) | p<0.001 |
| 40-59 | 64.7% (n=866) | 65.9% (n=2304) | p=0.713 |
| 60 and above | 26.9% (n=361) | 12.2% (n=425) | p<0.001 |
| **BMI-wide distribution** | | | |
| Normal | 4.7% (n=63) | 8.3% (n=290) | p<0.001 |
| Overweight | 20.8% (n=279) | 28.2% (n=987) | p<0.001 |
| Mildly obese | 30.3% (n=406) | 30.5% (n=1065) | p=0.944 |
| Moderately obese | 23.2% (n=311) | 18.6% (n=651) | p<0.01 |
| Severely obese | 20.9% (n=280) | 14.4% (n=503) | p<0.001 |
| **Blood pressure** | | | |
| Age groups (in years) |  |  |  |
| 20-39 | SP = 144.4019.86 | SP=123.4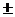12.2 | p<0.001 |
|  | DP = 93.40 12.16 | DP=80.3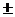9 | p<0.001 |
| 40-59 | SP=147.33 20.03 | SP=127.0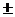15.8 | p<0.001 |
|  | DP=92.50 11.82 | DP=80.7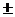9.1 | p<0.001 |
| 60 and above | SP=153.25  21.00 | SP=134.7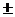18 | p<0.001 |
|  | DP=8811.00 | DP=80.1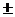9.0 | p<0.001 |
| **Mean BMI in kg/m^2^** | | | |
| Age group (in years) |  |  |  |
| 20-39 | 38.3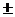7.3 | 34.2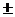7.3 | p<0.001 |
| 40-59 | 35.3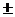6.8 | 32.9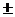6.3 | p<0.001 |
| 60 and above | 32.6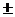6.5 | 31.3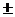5.9 | p<0.01 |
| **Mean onset age of T2DM in years** | | | |
| Male | 51.7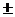11.9 | 46.7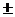11.3 | p<0.001 |
| Female | 54.5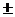10.50 | 47.9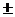10.8 | p<0.001 |
| **Mean onset age of hypertension in years** | | | |
| Male | 47.5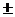11.7 | -- | NA |
| Female | 50.3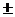10.3 | -- | NA |

^@^, The mean values presented in the previous two columns are compared using t-test.
